# An Innovative Technique for Identification of Missing Persons in Natural Disaster Based on Drone-Femtocell Systems

**DOI:** 10.3390/s19204547

**Published:** 2019-10-19

**Authors:** Roberta Avanzato, Francesco Beritelli

**Affiliations:** Department of Electrical, Electronic and Computer Engineering, University of Catania, 95125 Catania, Italy; roberta.avanzato94@hotmail.it

**Keywords:** drone/UAV system, femtocell, reference signal received power, mobile terminal classification algorithm, positioning algorithm, 4G radio technologies (LTE)

## Abstract

The recent development of the IoT (Internet of Things), which has enabled new types of sensors that can be easily interconnected to the Internet, will also have a significant impact in the near future on the management of natural disasters (mainly earthquakes and floods) with the aim of improving effectiveness in research, identification, and recovery of missing persons, and therefore increasing the possibility of saving lives. In this paper, more specifically, an innovative technique is proposed for the search and identification of missing persons in natural disaster scenarios by employing a drone-femtocell system and devising an algorithm capable of locating any mobile terminal in a given monitoring area. In particular, through a series of power measurements based on the reference signal received power (RSRP), the algorithm allows for the classification of the terminal inside or outside the monitoring area and subsequently identify the position with an accuracy of about 1 m, even in the presence of obstacles that act in such a way as to make the propagation of the radio signal non-isotropic.

## 1. Introduction

The possibility of new technologies able to detect the possible presence of missing persons involved in a calamitous event such as an earthquake or a flood is of great interest for civil protection organizations. The recent development of the IoT, which has enabled new types of sensors that can be easily interconnected to the Internet, will also have a significant impact in the near future on the management of natural disasters (mainly earthquakes and floods) with the aim of improving effectiveness in the research, identification, and recovery of missing persons, and therefore increasing the possibility of saving lives [1,2,3,4,5].

**Figure 1:**
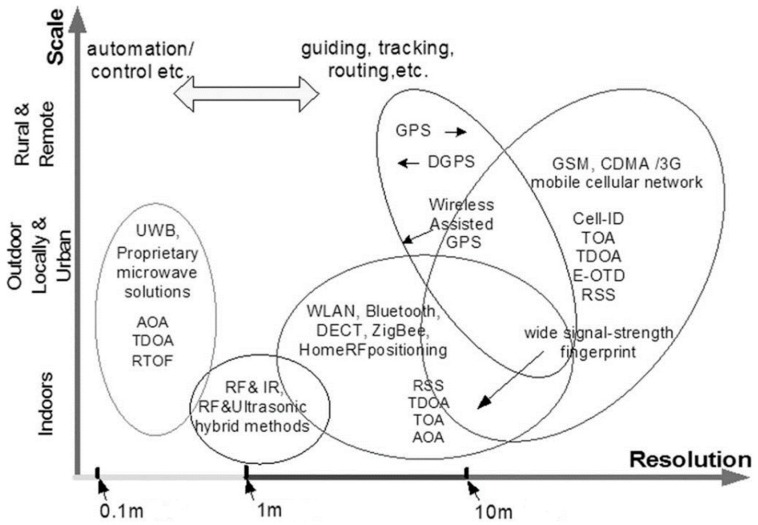
Currently existing wireless localization techniques.

In this paper, more specifically, an innovative technique is proposed for the search and identification of missing persons in natural disaster scenarios by employing a drone-femtocell system and devising an algorithm capable of locating any mobile terminals in a given monitoring area. It is a well-known fact that in the hours following a calamitous event, traditional radio base stations (BTS, base transceiver station) are very likely to be out of service or that, due to the attenuation of the radio frequency (RF) signal as the result of the rubble, the terminal no longer has regular coverage by the BTS as it loses the connection and interrupts the service. The idea of being able to create a cover through the presence of a femtocell on board a drone, which can then move freely and approach the user terminal, would give the possibility to locating any mobile terminals that may still be working under the rubble. In particular, through a series of power measurements based on the reference signal received power (RSRP) [6], the algorithm allows for the classification of the terminal inside or outside the monitoring area and subsequently to identify the position with certain accuracy, even in the presence of obstacles that act in such a way as to make the propagation of the radio signal non-isotropic. Figure 1 shows the current wireless localization techniques in the scale/resolution plan [7]. The area of the graph with the resolution of a meter and in an outdoor staircase (including rural and remote) is not currently managed and covered by any technique.

Some main radiolocalization methods are distinguished:

- Signal strength based (SSoA);
- AOA (DOA/DF) (Angle Of Arrival/Direction Of Arrival/Direction Finding);
- TOA (Time Of Arrival);
- TDOA (Time Difference Of Arrival);
- Hybrid techniques (a combination of two or more of the previous ones).

For the given scenario, the RF signal was considered and, in particular, among the various RF signals (BT—Bluetooth, WiFi—Wireless Fidelity, RFiD—Radio-Frequency Identification, etc.), the 2G/5G signal emitted by a mobile terminal has been singled out due to the power received from the femtocell to which it is attached. The first results of the tests, carried out with 4G systems, indicate that the proposed approach allows a geolocation with a more than adequate precision for the purposes of civil protection application scenarios.

The rest of this paper is organized as follows. Section 2 briefly summarizes the main localization and positioning techniques of mobile terminals, followed by Section 3, which illustrates the operating principles of LTE technology (i.e., architecture, communication channels, parameters used); Section 4 describes the hardware and software used; Section 5 highlights the proposed technique, which is divided into two phases: the classification phase and terminal localization phase; Section 6 outlines the technique used for the classification of terminals inside or outside the monitoring area; Section 7 characterizes the technique used for locating the terminal within the monitoring area without route optimization; Section 8 depicts the testbed and the various performance tests; and the last section is devoted to our conclusions.

## 2. State of the Art

The localization of a mobile terminal by RF signal is a topic often addressed in the literature [8,9]. In fact, there are several studies that have dealt with the localization of terminals, which are found in disaster areas or more generally in the presence of obstacles, through the analysis and measurement of radio signals.

In particular, in [10], the authors studied two different types of attenuation of the RF signal. The first study regarded the effect of the human body on the propagation of the signal. The result of this study led to the conclusion that the human body attenuates the radio frequency signal by an average of 20 dB. Subsequently, the attenuation of the signal due to the presence of rubble and, therefore, of different types of materials was studied, considering a signal at 1800 MHz. The results of this study show that, in a post-earthquake disaster scenario, the losses, compared to free space, are 13 dB greater than the losses that occur in an indoor environment, which are equal to about 5 dB.

In [11], the frequency response of the radio channel was studied for different frequency ranges and the signal attenuation was measured for two types of material: ceramic and brick. First, the 1.8 GHz attenuation was about 4.5 dB more than 900 MHz in the case where the obstacles were arranged evenly around the receiving antenna, and 17.5 dB more in the case where the obstacles were arranged in a less uniform way around the antenna. This implies that a higher frequency signal suffers more attenuation and that the obstacles placed in an irregular manner induce even more attenuation compared to the case in which they are arranged in a more uniform manner.

More recently, the growing need to connect and cover places affected by natural disasters has led to the commission of multiple studies concerning the use of drone-femtocell systems as an alternative to the classic radio base stations when these are out of service [12,13,14]. In these papers, in particular, new solutions were proposed to guarantee coverage and connection to users engaged in rescue operations. In [15], on the other hand, the possibility of identifying the mobile device when in an indoor environment using femtocells was illustrated by using hybrid methods based on the measurement of downlink and uplink signal strength and on the time of arrival (TOA).

## 3. Long Term Evolution Technologies

Long Term Evolution (LTE) stems from the need to support the growing demand for intelligent and secure connectivity, to have an ever increasing speed of access to the Internet, and to ensure high levels of QoS (Quality of Service) in accessing increasingly demanding services in terms of bandwidth.

**Figure 2:**
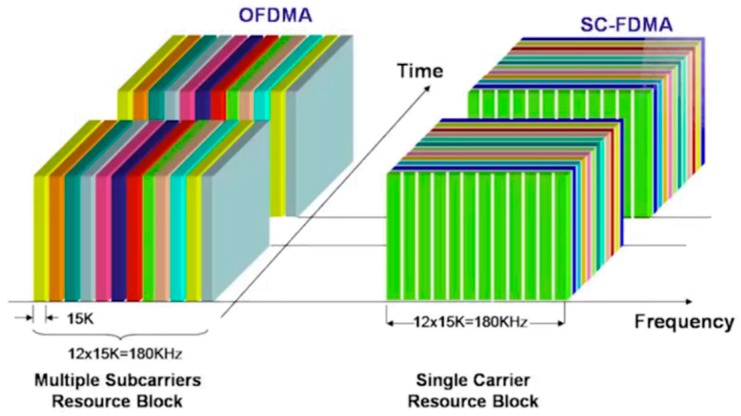
4G radio access techniques.

As far as radio access is concerned, the fourth generation system is characterized by the use of innovative techniques that allow for reaching high speeds of throughput and high performances of spectral efficiency (i.e., ratio between information speed and occupied bandwidth). Figure 2 shows the radio access technique:

- OFDMA (orthogonal frequency division multiple access) is a technique used for the downlink based on OFDM (orthogonal frequency division) modulation and works by assigning each user a subset of subcarriers in which the available band is divided for a determined interval of time, enabling a high data rate while maintaining a high level of robustness against interference. Thanks to its orthogonality property, an overlap between the spectra can be tolerated, thus allowing more information to be transmitted in the same band.
- For the uplink, we used the SC-FDMA (single carrier frequency division multiple access), which allows us to have a small peak/average power ratio, thus ensuring a high efficiency of power dissipated in the cell phones (a very important factor as it affects battery life).

**Figure 3:**
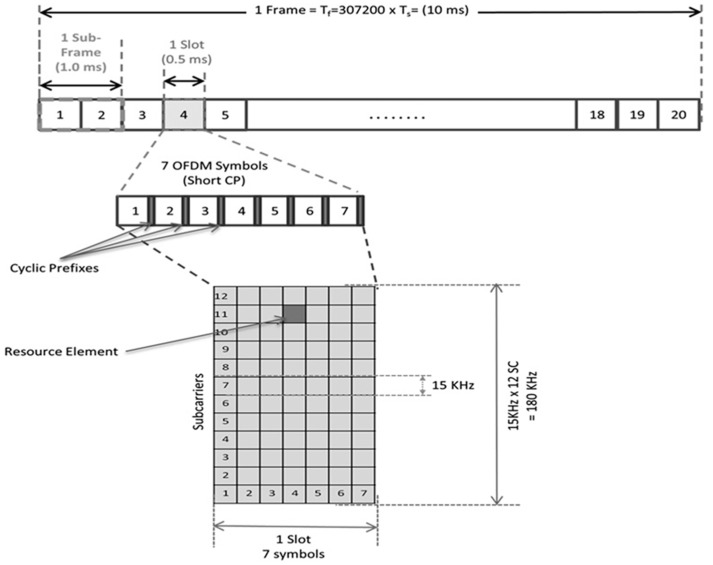
LTE frame structure.

Figure 3 shows the LTE frame structure. It has a duration of 10 ms and carries a defined set of sub-frames of 1 ms, which is called the TTI, the transmission time interval, where the transmission parameters are updated every TTI. A sub-frame contains two slots of 0.5 ms duration, which is formed by several OFDM symbols.

The basic unit is a Resource Element (RE), which extends over a subcarrier symbol. Each resource element usually carries two, four, or six bits of the physical channel, depending on whether the modulation scheme is QPSK, 16-QAM, or 64-QAM.

The resource elements are grouped in Resource Blocks (RBs), each of which extends over 0.5 ms (one slot) of 180 kHz (12 sub-carriers).

**Figure 4:**
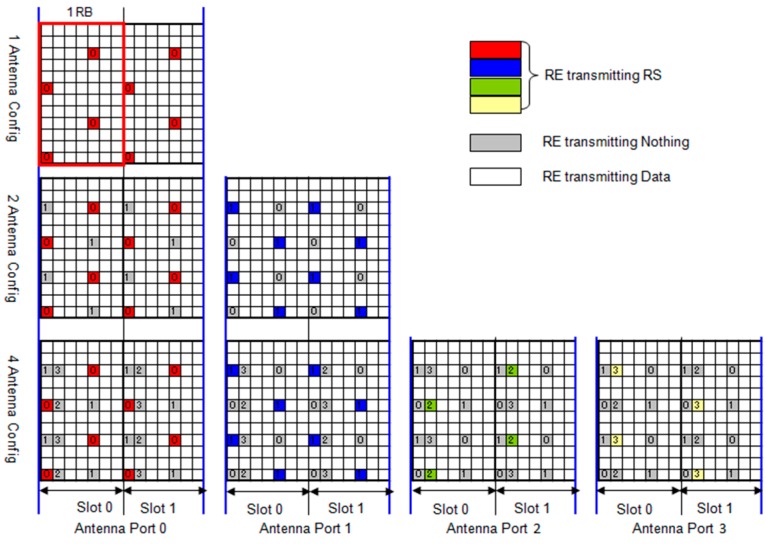
Reference signal mapping in the resource blocks.

In the Figure 4 it’s possible see how the Reference Signal (RS) is transported on a single resource element, which is important as they allow for estimates to be made on the channel by the terminal. In the time domain, two OFDM symbols per slot carry a reference symbol. The mobile terminal measures the power of the received signal from the RS and considers it as the transmission power in downlink of the eNodeB.

There are two types of reference signals:

1. Cell specific reference signal: every sub-frame is transmitted and extends across the entire operating band;
2. User Equipment (UE) specific reference signal: this reference signal is transmitted within the RBs assigned only to a specific UE, when beamforming is adopted.

In a cellular network, when a mobile terminal moves between cells and has to perform cell selection/reselection and handover operations, the power and signal quality of the neighboring cells must be measured. In a LTE, a UE measures the following two parameters starting from the reference signal, reporting them to the eNode:

- Reference Signal Received Power (RSRP);
- Reference Signal Received Quality (RSRQ).

From these two indices, the eNB goes back to the Reference Signal Strength Indicator (RSSI) as the UE does not report to the eNodeB.

The RSRP is a linear average of the power contributions (in Watts) of the resource elements carrying the cell specific reference signal in the considered measurement band (N resource blocks).

The RSRP, typically between −44 and −140 dBm, represents a good parameter of the power measurement of a specific sector excluding noise and interference from other sectors. The average RSRP values were around −75 dBm, when the UE was near an LTE station, and −120 dBm near the edge of the cell coverage area [16,17].

## 4. Tools

The following hardware and software tools were used for the simulations and laboratory tests:

- Qucell 4G/LTE Small Cell;
- Accuver XCORE/XIMS;
- GMON; and
- GeoDevice

**Figure 5:**
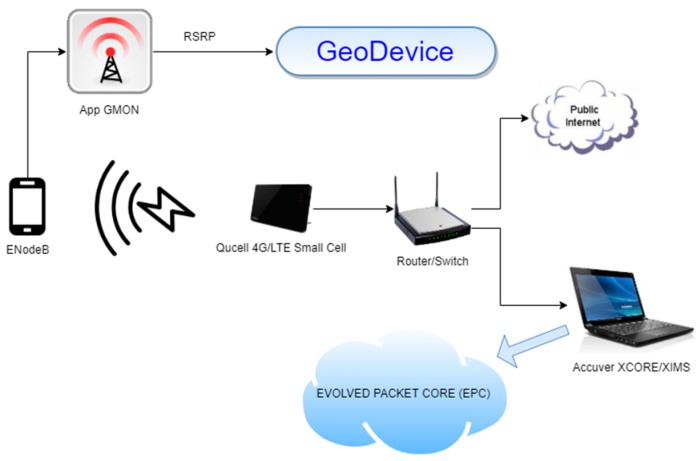
Hardware and software interconnection.

Figure 5 shows how the hardware devices and software tools were interconnected for measurements on the field carried out at the laboratory. The results of these tests are described in Section 8.

### 4.1. Qucell 4G/LTE Small Cell

**Figure 6:**
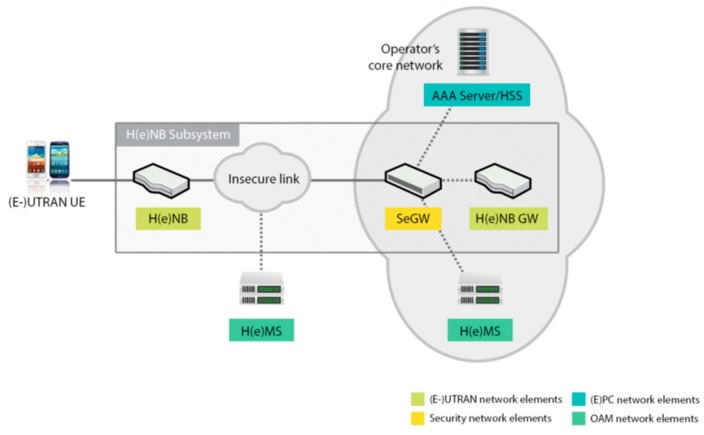
Connection diagram for the LTE Small Cell and Evolved Packet Core (EPC).

The femtocell is a small base station of limited radius coverage and low power designed for home or small business use. In particular, the Qucell LTE Small Cell was used for the test beds in this paper. It is also called HeNB (Home evoluted Node UTRAN B), and has the function of wireless connection with the UE (user equipment) to process packets with the LTE air standards between the EU and EPC. The LTE Small Cell has the interaction of the HeNB function with the Femto GW server (Gateway), SeGW (Security Gateway), and HeMS (HeNB Management System). The Small Cell (HeNB) is connected to the SeGW and, through the authentication process, creates an IPSec connection between the Small Cell and SeGW with which communication is activated. At the end of the authentication process, Small Cell is connected to the HeNB GW (Femto GW), ready to serve the services by communicating with the MME (mobility management entity). The process of the setup, management, alarm, statistics, etc. is managed through the HeMS Server, which has a connection with the Small Cell through the IP network [18]. The logical connection scheme for the LTE Small Cell and the Core Network are shown in Figure 6.

The hardware and software specifications [19] of the femtocell are described in Table 1 below:

### 4.2. Accuver XCORE/XIMS

Accuver XCORE / XIMS is software that implements EPC network functions in a PC and provides tests and the environment for E-UTRAN equipment: eNodeB or Home eNodeB. The EPC network includes MME (S1-MME) functions for mobility management and the EU-User Equipment session, S-GW (S1-U) functions for IP routing, Home Subscriber Server (HSS) functions for managing subscriber information, and the PCRF functions for QoS [20].

**Figure 7:**
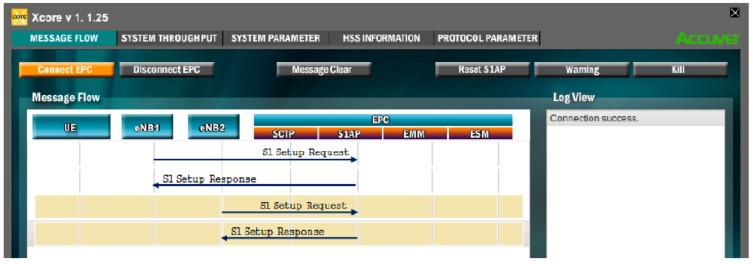
Message flow interface between eNB and EPC.

Figure 7 shows the interface relating to the flow of messages exchanged in the connection between eNodeB and EPC.

### 4.3. GMON

G-MoN is an application made in Android to control the signal level of a Wi-Fi network and to obtain very complete and detailed analysis of a 2G/3G/4G network. When running the application, apart from basic settings, G-MoN offers several options in its graphical interface such as:

- “2G/3G/4G”: presents information and parameters relating to the mobile radio network (“CID”, “LAC”, “RXL”, “RSRP”, “RSRQ”, etc.). In particular, the information given by “RSRP” and “RXL” serves as the basis for the localization algorithm proposed in this paper;
- “Wi-Fi”: presents all the data concerning the wi-fi connection (“SSID”, “CH”, “RXL”, etc.);
- “Cell History”: contains what you need to know about the coordinates (latitude “LAT” and longitude “LON”), precision (“ACC”), and altitude (“ALT”). In this case, obviously, the GPS must be enabled.

### 4.4. GeoDevice

In order to carry out a series of simulations of the scenario and the techniques proposed in this paper, a specific tool called GeoDevice was designed and implemented. The software implements a graphic engine to meet the project specifications. The graphic library adopted (low level) was OpenGL, which ensures extreme flexibility, not only in terms of graphic performance, but also (and above all) cross-compiling on Linux, Windows, and Mac.

The structure of the engine is based on a series of low-level operating classes designed to reconstruct the three-dimensional primitives needed in the rendering phase. The rendering is made by objects, therefore it is easy to extend the graphic functionalities defining new classes and leave the reconstruction operations to the aforementioned low level classes. The engine also implements a moving camera that allows for the exploration of the three-dimensional scenario in virtual reality.

The tool implements the:

- Classification phase, which allows the classification of the terminals within or outside the various monitoring areas, considering, or not, the additional attenuation;
- Localization phase, which allows the insertion of the scene in question and the data obtained from the measurements in the field, subsequently applying the localization algorithm of the mobile terminal.

Regarding the “classification phase”, the tool provides for the insertion of mobile terminals in one or more monitoring areas, simulates the power received from the terminals when the femtocell runs along the perimeter of the area, and generates the polar diagram determining if the terminals are classified within or outside the monitoring area.

As for the “localization phase”, the tool is meant to outline a scene, which, for the study carried out in this paper, corresponds to a building of a certain length, height, and depth. Once the scene has been defined, it is possible to load the data through a file with the “.json” extension. The data represent the power values measured at precise coordinates defined a priori. Once the data are loaded, the tool automatically proceeds to apply the algorithm for locating the terminal, adopting one of the three methods described above, based on the “power wall” created.

## 5. Proposed Technique

The localization technique of a proposed mobile terminal uses 4G LTE technology at a frequency of 1805–1880 MHz. A femtocell positioned on board a drone hooks the terminal univocally. For software limitations due to the femtocell in use, it is not possible to extrapolate the power value that it receives from the common control channel of the smartphone. For this reason, in our tests, the power value will be read by an app (GMON) installed on the mobile terminal.

Due to a more accessible femtocell, from the software point of view, we have the security (due to the LTE technology standards) that the RSRP parameter can be read subsequently by the femtocell.

In our tests, the highest power value was associated with a dark red color, while the lowest measured power value was associated with a dark blue color.

The proposed algorithm can be divided into two phases:

1. Classification of the internal/external terminals in the monitoring area; and
2. Localization of the terminal within the monitoring area.

**Figure 8:**
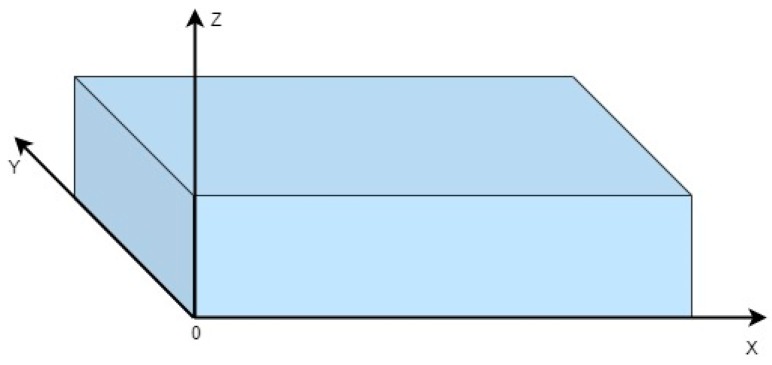
Outline of the monitoring area.

In the first phase, the focus is on discriminating the mobile terminals inside or outside the considered monitoring area. This is characterized by one or more devices under the rubble, so it is probably represented by an area of collapsed buildings. The proposed technique involves modeling the monitoring area, for simplicity, incorporated within a three-dimensional parallelepiped, of a given height h, thickness y, and depth x, shown in Figure 8. The x and y parameters represent the dimensions of the area to be monitored, while with h, the rubble height is indicated within the monitoring area, or the minimum height of the floor above the monitoring area to which the drone can fly without encountering obstacles.

### 5.1. Mobile Terminal Classification

In a generic scenario characterized by multiple monitoring areas, the first step is to verify the possible presence and the number of mobile terminals for each monitoring area to select the areas of interest and immediately conduct the search by civil protection bodies.

**Figure 9:**
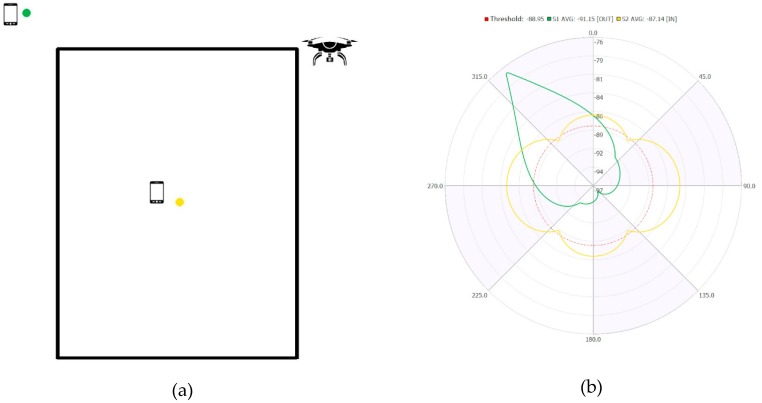
(**a**) i-th monitoring area; (**b**) Polar diagram of the measured power.

Assuming an ideal scenario, in the presence of free space, the proposed classification technique involves having the drone-femtocell system go around the entire perimeter of the i-th monitoring area, measuring on the femtocell, with a step chosen a priori, the value of RSRP power received from the terminal. Figure 9 shows an example of a polar graph of the power levels obtained in the hypothesis of an internal terminal (orange curve) and an external one (green curve) to the monitoring area.

The circumference in blue represents the value of the discrimination threshold to distinguish whether the related terminal is external or internal to the monitoring area. In particular, the threshold is calculated considering the mean $RSRP_{Threshold}$ of the power measurements obtained by the drone-femtocell system that runs along the entire perimeter of the monitoring area, with respect to a mobile terminal placed in a corner of the monitoring area.

Given a mobile terminal, the relative average power on the perimeter of the monitoring area can be calculated, where the classification criterion is based on the following relation:

- For $RSRP_{t}^{AVG}<\;RSRP_{Threshold}$, the device is classified as OUT;
- For $RSRP_{t}^{AVG}>\;RSRP_{Threshold}$, the device is classified as IN.

The algorithm is applied to all the terminals that can engage the femtocell, keeping in mind that this allows for their unique identification to be determined iteratively from the monitoring area n = 0 to the area n = N. It is important to point out that the power calculation refers to values obtained through the Friis formula (assuming a free space):

(1) $$P_{R}=\;P_{T}G_{T}G_{R}{\left(\frac{\lambda }{4\pi R}\right)}^{2},$$

where

- PR is the signal strength that the terminal receives, which in LTE technology is called RSRP;
- GT and GR are the gain of the antenna in transmission and in reception, respectively;
- PT is the signal strength transmitted by the femtocell; and
- R is the distance between the femtocell and the mobile terminal (a value that is known in the simulation phase, because it is chosen a priori).

To verify the reliability of this technique, it is necessary to apply it in real scenarios, where the terminals (of all the monitoring areas) are subject to the non-ideal conditions of the system, even when they are in free space, and even worse, when they are covered by rubble with more or less density. To analyze this more complex scenario, several hypotheses have been formulated.

The possible cases to be studied are the following:

- Attenuation from non-ideal free space: the non-ideality is due to the presence of the soil, to the height of the Tx and Rx antennas, multipath, positioning of the smartphone, and the femtocell;
- Uniform attenuation due to a single type of material;
- Not uniform attenuation due to a single type of material; and
- Uniform attenuation (various materials).

In this paper, the first case was taken into consideration, analyzing the results even when they varied:

- the height, width, and depth of the monitoring area;
- the height of the drone; and
- the height of the terminals inside and outside the monitoring area.

The algorithm, proposed and described in Section 6, takes into account the previous points and compares the performance of the results obtained from the simulation.

### 5.2. Mobile Terminal Localization

Once the presence of one or more mobile terminals within a monitoring area has been verified, the second phase aims to estimate the position of the individual device. In fact, the algorithm repeats the analysis for each terminal, thanks to the possibility that the femtocell offers us to measure the power values unambiguously for each individual terminal.

**Figure 10:**
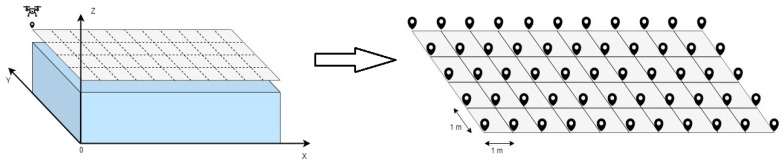
Rectangular grid of the measurement points.

A rectangular or cross-linked grid of uniformly distributed measurement points is considered to be placed exactly above the surface of the parallelepiped enclosing the monitoring area, where the drone, by moving, makes various measurements of the received signal strength. An example is shown in Figure 10, where there is a 5 × 11 m grid and the real position of the terminal is represented by the square in blue.

**Figure 11:**
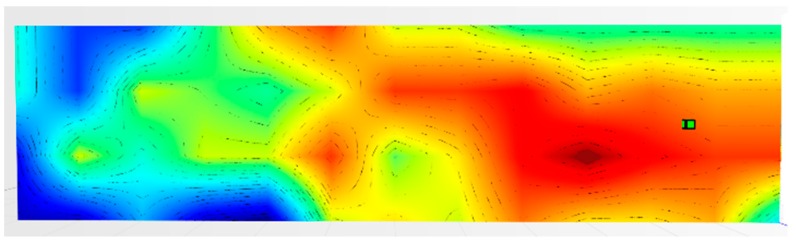
“Power wall” of the values measured in the grid points.

The power received by the terminal was subsequently mapped and interpolated, creating what has been called a “power wall”. An example of the latter is shown in Figure 11, which is the result of the power measured in the grid points in Figure 10, interpolated and mapped. The terminal is in the position marked by the green cube:

Starting from the data of the power measurements contained in a “power wall”, the proposed algorithm for estimating the position of the terminal was based on an aggregation of the following three estimation methods:

- “Method of proximity” to the highest power value;
- “Weighted distance method”; and
- “Center of gravity method”.

The first estimates the position of the terminal in the position in which it has the highest power value. This algorithm is very efficient in scenarios where the terminal is in free space or when the rubble has a uniform distribution and attenuation over the entire area. In scenarios where the area does not have a uniform type of attenuation, this algorithm appears to be poorly suited to the context.

For this reason, the “weighted distance method” was introduced, which identifies the relative minimum points of the power levels. In fact, for certain types of material attenuation, the RF signal source points may be strongly attenuated, but in their surroundings still radiate the signal and, therefore, define the so-called “volcano mouths”, which, if recognized, allows for the mobile terminal that is in the vicinity of these to be determined and not in the points where the power level is the maximum.

Finally, if the “power wall” shows three peaks of maximum power and no relative minimum, the “center of gravity method” is activated, which precisely estimates the terminals at the point defined by the formula of the center of gravity, where the three points that define the triangle are precisely the peak points of maximum power. In particular, the three algorithms will be explained more specifically in Section 8.

**Figure 12:**
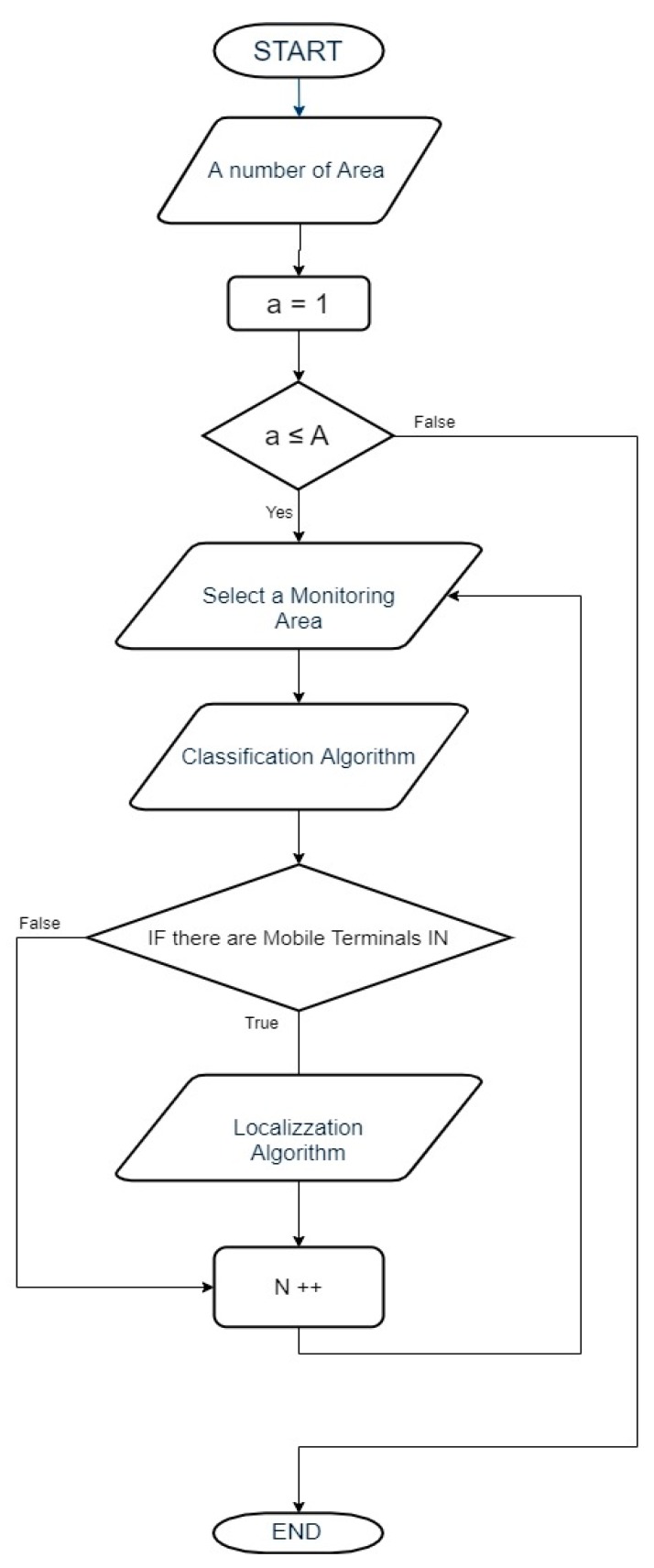
Flowchart of the proposed technique.

In general, the algorithm can be represented using the following flow chart (Figure 12):

## 6. Classification Algorithm

In this section, we describe the classification algorithm, which, as above-mentioned, allows us to understand how many terminals are inside or outside the monitoring area. Considering the complexity of the analysis to be performed, certain hypotheses were introduced:

- The path followed by the drone, with the femtocell on board, is along the perimeter of the surface of the parallelepiped (which represents the area to be monitored) in steps of one meter;
- It is assumed that the femtocell has an isotropic propagation and an infinite coverage radius, thus allowing the coupling of all the internal and external terminals to the monitoring area; and
- The height of the drone, in meters, is equal to:
  (2) $$H_{D}=H_{R}+0.5,$$
  where
  $H_{D}$ is the height of the drone with the femtocell on board; and
  $H_{R}$ is the maximum height level of the rubble.

In an initial phase, we exclusively used the average power measured for terminal discrimination, which is calculated as follows:

(3) $$AVG=\frac{1}{N}\overset{N}{\underset{i=0}{\sum }}P_{R}{}_{i},$$

where N is the number of measurement points taken on the perimeter of the monitoring area and $P_{R}{}_{i}$ is the simulated power value or the one obtained from field tests.

In order to simulate the power value of $P_{R}{}_{i}$ of the i-th measurement point, the Friis formula (see Equation (1)) is used.

The choice to use only the power average parameter makes the system not very stable to the attenuation and multipath effects, for this reason, two other parameters were added:

- Normalized variance; and
- First autocorrelation coefficient.

For the calculation of the first parameter, a preliminary step is carried out. It works by taking the lowest power value (remember that the power in dBm has a negative sign), calling it $P_{R}{}_{min}$ and normalizing the other power values with respect to this, with the following formula:

(4) $$NVAR=\frac{1}{N}\overset{N-1}{\underset{i=0}{\sum }}{\left(P_{norm}{}_{i}-AVG\right)}^{2},$$

where $P_{norm}{}_{i}$ is

(5) $$P_{norm}{}_{i}=\frac{P_{R}}{P_{R}{}_{min}},$$

The calculation of the second parameter, first autocorrelation coefficient, is given by the following:

(6) $$\rho_{1}=\frac{CORR_{1}}{CORR_{0}},$$

where

(7) $$CORR_{k}=\overset{N-1}{\underset{k=0}{\sum }}\left\{\;\frac{1}{N}\overset{N-k-1}{\underset{t=0}{\sum }}\left[\left(P_{R}{}_{t}-AVG\right)\cdot \left(P_{R}{}_{t+k}-AVG\right)\right]\right\}$$

To take into account the type of femtocell and the coverage levels offered, the algorithm is based on a Friis formula “compensated”. The compensation lies in adapting the value of the variance obtained from the measurements in the field with that of the measurements obtained from the simulator that uses the Friis formula in free space. The balanced Friis formula, therefore, can be derived from the following formula:

(8) $$P_{R}^{Comp}=\;\alpha P_{R}+\left(1-\alpha \right)\bar{P_{R}}\;,$$

where α=0.52 (the value was chosen based on the measurements made in the field) and $P_{R}$ is the power of Friis’s formula, while $\bar{P_{R}}$ is the average of the values given by the Friis formula.

To this compensated formula is added an error with Gaussian distribution (*Eg*), which represents the non-ideality of the system (in particular, the not perfectly isotropic propagation and the measurement error). The procedure underlying the generation of the Gaussian error is the following:

- A test of real measurements is considered, where there are four terminals: two inside and two outside the monitoring area. The four terminals measure a power value (RSRP), respectively, as the femtocell position changes along the perimeter of the monitoring area;
- In the same conditions, the measurements are generated by the simulator for each terminal;
- The difference is made between the values generated and those measured for each terminal (obviously with the same position in the monitoring area);
- The standard deviation of the differences is calculated;
- An average of the four calculated standard deviations is calculated; and
- The resulting value corresponds to the range of the Gaussian error, which in our tests goes from [−1.39,1.39].

The final formula for generating power values, in non-ideal conditions, in the simulator is derived from the following:

(9) $$P_{R}^{f}=\;P_{R}^{Comp}+\;E_{g}$$

Section 6.1. describes the classification algorithm that uses a single threshold, called the critical threshold, calculated by assuming the terminal in a corner of the perimeter, and, moreover, the performances are evaluated by varying some parameters. Similarly, in Section 6.2, the algorithm is described using two thresholds, one positioned in the corner of the monitoring area (i.e., the critical threshold), and the other is positioned in the center of the monitoring area, the latter being defined as the “threshold centroid”.

### 6.1. Single Threshold Method

In the first phase, the threshold was considered, assuming a mobile terminal placed on the ground (0 m), in correspondence with an edge of the monitoring area.

The parameters used for the implementation of the classification algorithm are:

- Power average;
- Normalized variance; and
- First autocorrelation coefficient.

The algorithm is defined by the combination of the three parameters described above. If the classification parameters of the first terminal are smaller than the second, then the output to the logic chain will have a Boolean variable set to TRUE, otherwise, to FALSE.

**Figure 13:**
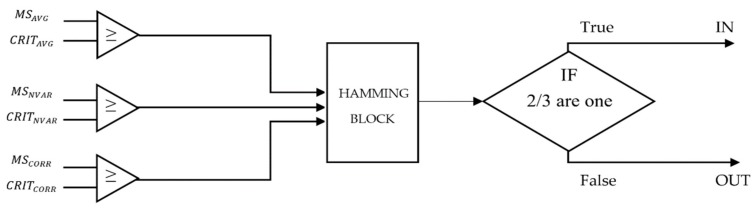
Block diagram of the critic threshold based on the classification algorithm.

Figure 13 shows the block diagram of the classification algorithm:

If the output of the AND gate is TRUE, this means that the terminal is correctly classified IN, otherwise, if the output is FALSE, the terminal is classified as OUT.

Performance evaluation takes place with the following parameters:

○ Drone and rubble quota: please note that these two parameters are linked by the Equation (2) expressed above;
○ Monitoring area size.

Before proceeding with the performance evaluation, it is important to introduce the definition of some terms:

○ “Monitoring Area”, is the area where it is necessary to check how many terminals are inside and how many are outside;
○ “Critical Zone”, is the area outside the monitoring area where it is possible to find false positives such as terminals that are classified as IN, but which, instead, are OUT;
○ “Safe Zone”, which represents a security zone around the monitoring area that is assumed to have no terminals; and
○ “Rubble Level”, which is the maximum level of the rubble in a disaster scenario.

After defining these expressions, we proceed to the evaluation of the performance according to the parameters described above.

#### 6.1.1. Accuracy as the Amount of Rubble Varies from “Safe Zone” by 10%

The accuracy of the IN and OUT terminals is evaluated by introducing a “Safe Zone” of 10% around the “Monitoring Area” (i.e., 10% of the short side of the monitoring area). Furthermore, to take into account the radius of coverage of the femtocell, in the real case, is of finite value, we considered the possible presence of external terminals positioned up to 50 m from the monitoring area.

**Figure 14:**
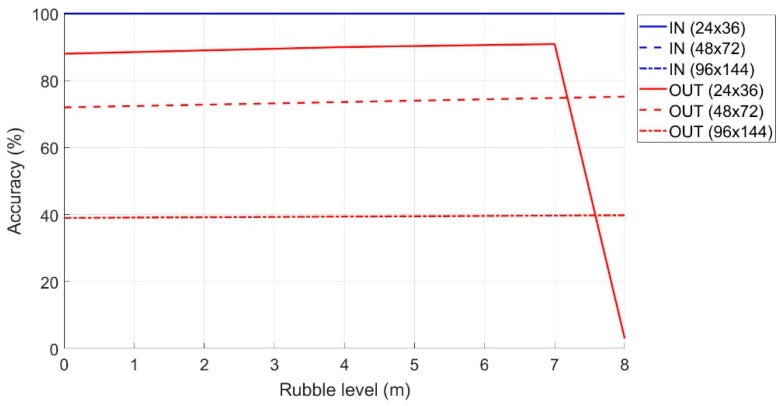
Accuracy in the classification of the IN and OUT terminals as the amount of rubble increases.

In Figure 14 is shown the accuracy in the classification phase when the rubble level varies. In terms of the percentage accuracy of the terminals correctly classified as IN and OUT, it is clear that for the different dimensions of the monitoring area, the accuracy of the IN was 100% (i.e., all the terminals that were IN were classified as such). Conversely, the accuracy of the OUTs (i.e., external terminals that are classified as such) is very variable, and this depends both on the size of the monitoring area and on the level of the rubble. The accuracy decreases quickly after a certain flight height of the drone (or rubble) due to the greater visibility that the femtocell has from the mobile terminals outside the monitoring area.

In general, both the performance related to the extension of the “Critical Zone” and those relating to accuracy were very low, for this reason, a second threshold terminal was introduced and placed at the center of the monitoring area at drone height. In this way, as we will see later, the performance will improve.

#### 6.1.2. Extension of the “Critical Zone” as the Drone Quota Changes

As already indicated in Equation (2), the hypothesis on the drone’s flight height is that it exceeds the maximum height of the rubble level by half a meter, so that the drone can move freely within the whole monitoring area.

**Figure 15:**
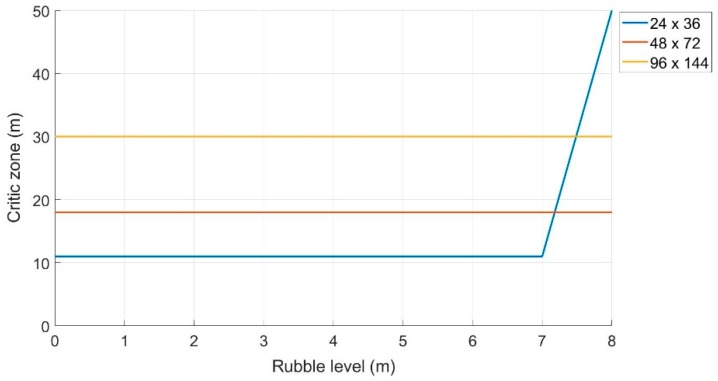
Variation of the “Critical Zone” as the amount of rubble increases.

As can be seen from Figure 15, the “Critical Zone” remains constant at 18 m and 30 m when the level of rubble varies for an area size of 48 × 72 (m^2^) and 96 × 144 (m^2^), respectively

While, in the case of the area of 24 × 36 (m^2^), the “Critical Zone” shows a sudden increase when the rubble exceeds 7 m in height. This means that the algorithm considers the mobile terminal as internal to the monitoring area, when in reality, it is positioned externally. These false positives lead to the increase of the “Critical Zone” by about 40 m, when the rubble level exceeds a certain threshold (in this case seven meters).

### 6.2. Method with Hybrid Thresholds

**Figure 16:**
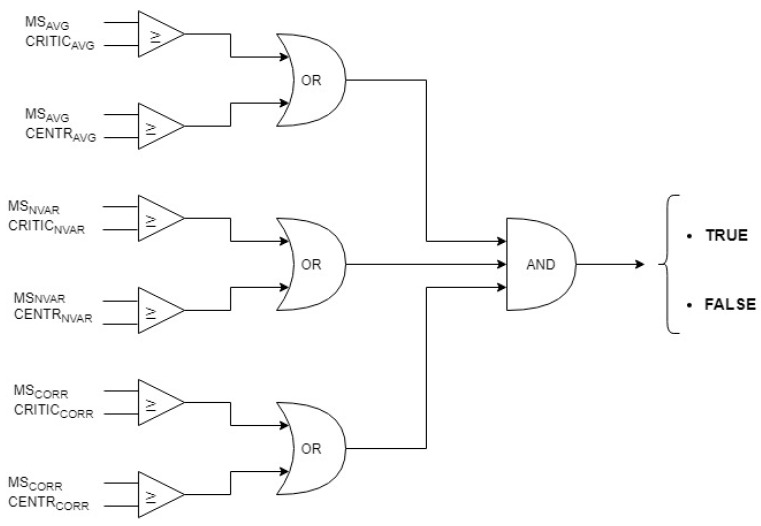
Block diagram of the hybrid threshold based classification algorithm.

This method has been implemented to increase the accuracy performance. The classification algorithm performs the calculation of the classification parameters by separately using the thresholds deduced from the analysis of the data relating to the two threshold terminals (critical and centroid) and, subsequently, applies a logical decision to establish whether the intercepted terminal is inside or outside the monitoring area. Schematically, we can represent the set of logical instructions as follows in Figure 16:

If at the end of the chain, the AND logic gate indicates TRUE, the terminal will be classified as IN, otherwise it will be classified OUT.

The flowchart in pseudo code is as follows:
**bool** cAVG = (AVG_T_ ≥ AVG_CRIT_) **or** (AVG_T_ ≥ AVG_CENTR_);
**bool** cNVAR = (NVAR_T_ ≥ NVAR_CRIT_) **or** (NVAR_T_ ≥ NVAR_CENTR_);
**bool** cCORR = (CORR_T_ ≥ CORR_CRIT_) **or** (CORR_T_ ≥ CORR_CENTR_);
**bool** c = (cAVG **and** cNVAR **and** cCORR);
**if** (c == true) {
   Mobile Terminal is IN
}
**else** {
   Mobile Terminal is OUT
}        
		


The variables “AVG_T_”, “NVAR_T_”, and “CORR_T_” refer to the parameters of the target terminal. The variables “AVG_CRIT_”, “NVAR_CRIT_”, and “CORR_CRIT_” refer to the threshold terminal at the critical point (the corner of the monitoring area) while the variables “AVG_CENTR_”, “NVAR_CENTR_”, and “CORR_CENTR_” refer to the threshold terminal in the centroid.

With this method, the performance of the classification algorithm was improved when compared to the previous method, however, there are still certain factors of non-ideality that affect its accuracy.

It is now possible to evaluate the performance with the new method as the parameters already described in the previous method vary and identify the differences and improvements.

#### 6.2.1. Extension of the “Critical Zone” as the Drone Quota Changes

**Figure 17:**
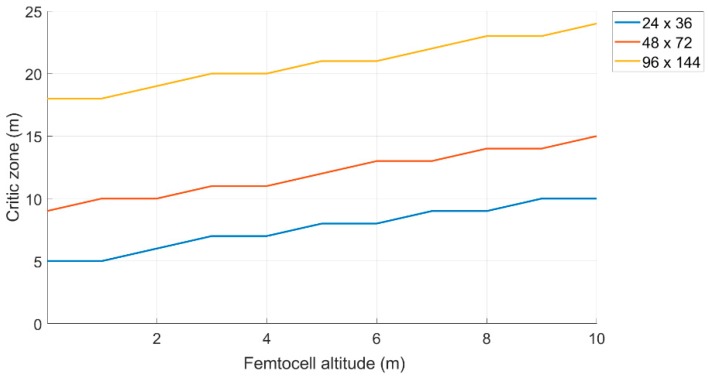
Variation of the “Critical Zone” to the increase of the femtocell altitude.

As can be seen from the Figure 17, compared to the previous method, the “Critical Zone” for each area appeared to be smaller in size, but grew slightly as the height of the rubble (and therefore of the drone) increased. In general, however, the performance improved, in fact, if the height of the rubble was 5 m, the area considered was 24 × 36 m, and the “Critical Zone” was about 7 m, while in the previous case, it was 11 m. Assuming that the size of the monitoring area and the level of the rubble is known, it is possible to determine the size of the “Critical Zone”, and therefore estimate a certain “Safe Zone”, which reduces the possibility of false positives (i.e., mobile terminals incorrectly classified as IN).

#### 6.2.2. Accuracy when Varying the Amount of Rubble

When the femtocell is positioned at the height of the rubble (therefore depending on the surrounding environment), the performance of the percentage accuracy relative to the OUTs depends not only on the actual dimensions of the “Monitoring Area”, but also on the position in which the internal and external terminals are positioned. As shown in the figure, the percentage accuracy of the IN is a straight line with a slight slope (about 1% is lost every 10 m) and suffers little from the influence on the femtocell share elevation. The situation is considerably different with regard to the percentage accuracy of the OUTs, which is significantly affected by the difference in altitude. In this regard, it is necessary to make an important clarification regarding what is meant by rubble quota:

○ The rubble quota (indicated as *H*_R_) identifies the maximum height of the rubble inside the “Monitoring Area”.
○ The OUT rubble dimension identifies the maximum height of the rubble surrounding the “Monitoring Zone” (i.e., those inside the “Critical Zone”).

**Figure 18:**
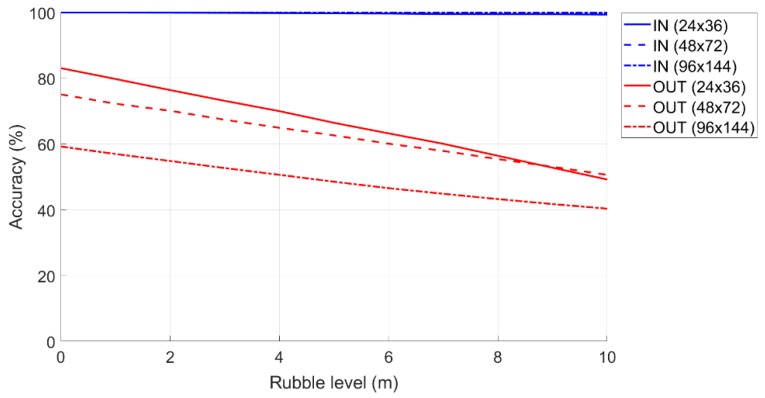
Accuracy in classification of the IN and OUT terminals as the rubble level increases.

In Figure 18, the maximum amount of rubble of 4 m was considered within the “Monitoring Area”, while the area surrounding the “Critical Zone” has rubble at a maximum height of 2 m, the percentage of accuracy IN will be identified in the graph from the blue curve to the altitude 4 m, while the accuracy percentage OUT will be identified in the graph from the red curve at a 2 m altitude.

#### 6.2.3. Accuracy when Varying the Amount of Rubble with the “Safe Zone”

To increase the percentage accuracy of the OUTs, a “Safe Zone” of 10% can be set up (calculated with respect to the short side of the “Monitoring Area”). In this way, a significant improvement can be identified in the accuracy performance of OUT (leaving unchanged those related to the classification of IN).

**Figure 19:**
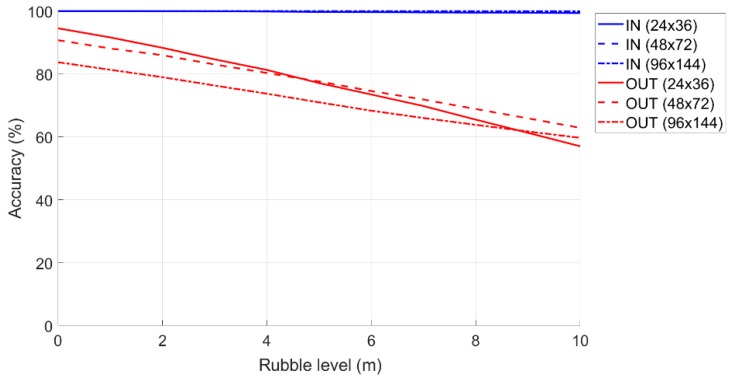
Accuracy in the classification of the IN and OUT terminals as the amount of rubble level increases with the “Safe Zone” by 10%.

Figure 19 shows the accuracy of the IN and OUT terminals when the rubble level increases when the “Safe Zone” is equal to 10%. Compared to the first case, the performance of the terminals classified as OUT, for all the dimensions of the areas, turned out to be greatly improved. In fact, if we were at a rubble quota of 5 m, for the 96 × 144 m area, we had an accuracy of about 70% when compared to the previous case which was 40%.

### 6.3. Method with Hybrid Thresholds and Femtocell Height at one Meter from the Ground

In the previous subsections, an important hypothesis was that the femtocell is placed above the drone, where the latter flies at a certain height, given by Equation (2), along the perimeter of the surface of the monitoring area. In this subsection, we modified this hypothesis and considered that the femtocell was placed on a mobile robot and that the height that could be reached varied from one to two meters. The path will be along the walls of the parallelepiped.

The following are the performance scenarios with the new hypothesis.

**Figure 20:**
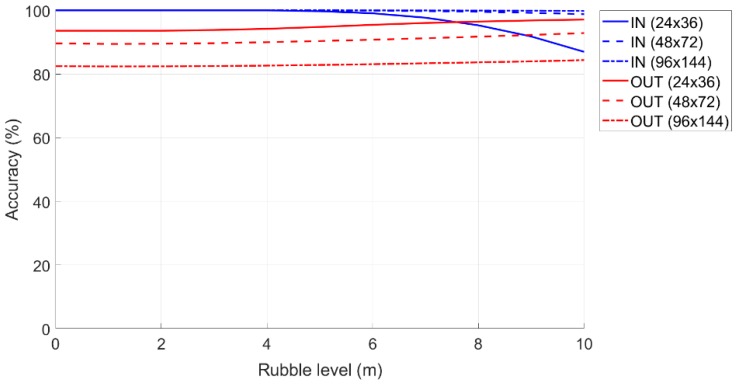
Accuracy in the classification of IN and OUT terminals as the amount of rubble increased and the femtocell was at a height of one meter from the ground.

When the femtocell is positioned at a man’s height (by hypothesis 1 m), therefore, independent of the surrounding environment, the performance of the percentage accuracy depends on the geometry of the “Monitoring Area” and the amount of rubble. However, in this case, the accuracy of OUT in percentage increases. Additionally, by considering a “Safe Zone” of 10%, the trend obtained was as follows:

Figure 20 shows the accuracy of the IN and OUT terminals when the rubble level increases and the femtocell is at height of one meter.

Comparing the two detection methods (at the rubble level and at a height of one meter), it can be observed that with the same simulation parameters, the femtocell method at a height of one meter was more efficient. The only clarification to make with regard to the decreasing trend of the IN accuracy was visible on the curve generated for a “Monitoring Area” with a size of 24 × 36 m. Furthermore, judging by the slight slope visible on the other two curves, one can speculate that a similar behavior will also be exhibited for the “Monitoring Area” for larger rubble dimensions.

## 7. Terminal Positioning Algorithm

Once the position of a mobile terminal has been classified as internal to the monitoring area, the next step is to estimate the exact positioning of each individual terminal within the selected area. In particular, three types of algorithms are illustrated in this second phase. The algorithm, which is based only on the method of proximity to the highest power value, as already mentioned, is not very efficient in more complex real scenarios.

In fact, due to different attenuation of the various materials around the terminal to be identified, it is likely that the mobile terminal receives more power along a lateral, and not orthogonal, propagation direction of the RF signal from the femtocell to the terminal, therefore, less power (and therefore greater attenuation) is detected in the area above the device and greater power in areas distant from the actual position of the mobile terminal. In this regard, an algorithm was designed to evaluate the position of the terminal, taking into account the presence of what we will call “mountains” and “lakes” that is, points where there are relative maxima and minima in the RF signal power values.

The concept around the definitions of “mountain” and “lake” is therefore based on the analysis of the monotony of isopotential surfaces extracted from the interpolated map of measured power levels. By grouping the surfaces and analyzing the trend of the power (from the internal to the external surface), it is possible to establish the direction of the power gradient. Depending on the direction taken, we defined:

- “Lake” when the innermost isopotential surface showed a growing monotonous tendency around a spatial position called the “peak of the lake”; and
- “Mountain” when the innermost isopotential surface showed a growing monotonous tendency around a spatial position called the “peak of the mountain”;

Before describing the operation of the algorithm, we introduced a series of definitions and formulas necessary for calculating the terminal estimate:

- $G_{max}$ is the maximum power value (dBm) detected in the entire matrix of points;
- $G_{min}$ is the minimum power value (dBm) detected in the entire matrix of points; and
- T is defined as “Earth level”, and is obtained by the following formula:
  (10) $$T=\frac{G_{max}\;+\;G_{min}}{2},$$
- $M_{peak}$ is the power (dBm) related to the maximum peak of the mountain;
- $L_{peak}$ is the power (dBm) related to the lake’s peak;
- $P_{M}$ is the position of the mountain’s peak;
- $P_{L}$ is the position of the lake’s peak; and
- $\delta_{M}$ is defined as “mountain weight” (i.e., the weight to be assigned to the global maximum point with respect to the “Earth” plan). This is defined by the following formula:
  (11) $$\delta_{M}=\frac{M_{peak}\;-\;T}{G_{min}-\;L_{peak}},$$
- $d_{LM}$ is the length of the segment that connects the peak of the “lake” with the peak of the “mountain”; and
- $P_{Norm}$ is the normalization power, and is obtained by the following formula:
  (12) $$P_{Norm}=G_{max}-G_{min},$$
- $D_{Norm}$ is the normalization distance, and is given by the diagonal of the considered rectangular surface. The formula is the following:
  (13) $$D_{Norm}=\sqrt{w^{2}+h^{2}},$$
  where w and h are the width and the height of the surface, respectively.

### 7.1. The Localization Algorithm Phases

The algorithm has three phases:

#### 7.1.1. Phase 1—Identification of “Lakes” and “Mountains” Using Contour Maps

The concept around the definitions of “mountain” and “lake” is based on the analysis of the monotony of isopotential surfaces extracted from the interpolated map of measured power levels. By grouping the surfaces and analyzing the trend of the power (from the internal to the external surface), it is possible to establish the direction of the power gradient. Depending on the direction taken, we defined:

- “Lake” when the innermost isopotential surface showed a growing monotonous tendency around a spatial position *P_Li_* called the “peak of the i-th lake”; and
- “Mountain” when the innermost isopotential surface showed a decreasing monotonous tendency around a spatial position *P_Mj_* called the “peak of the j-th mountain”.

#### 7.1.2. Phase 2—Filtering of Mountains and Lakes

The algorithm optionally involves the selection of mountains and lakes with higher power levels. In particular, if the FLAG of the filter is active, we filter “lakes” and “mountains” with a certain percentage, defined by the operator, with respect to the maximum peak power of the “mountain” and the minimum of the “lake”.

#### 7.1.3. Phase 3—Positioning Estimation Algorithm

- If the number of “mountains” is equal to zero, then the algorithm does not provide any result;
- If the number of “mountains” is equal to one, then the following elements must be evaluated:
  ○ If there are no “lakes”, the position is estimated by employing the proximity method;
  ○ If only one “lake” is found, the position is estimated by using the weighted distance method;
  ○ If several “lakes” are found, the “lake” with the lowest Mn value is selected (see Equation (17)), then the terminal is estimated by using the weighted distance method;
- If the number of “mountains” found is equal to two:
  ○ If no “lakes” are found, the position is estimated from the midpoint of the two “mountain” peaks;
  ○ If only one “lake” is found, the position is estimated using the distance criterion described below;
  ○ If the number of selected “lakes” is greater than 1, the “lake” with the lowest Mn value is selected (see Equation (17)), then the terminal is estimated using the distance criterion;
- If the number of “mountains” found is equal to three:
  ○ If there are no “lakes” the position is estimated using the center of gravity method;
  ○ If the number of “lakes” found is equal to one, the position is estimated using the distance criterion;
  ○ If the number of “lakes” found is greater than one, the “lake” with the lowest Mn value is selected (see Equation (17)), then the terminal is estimated using the distance criterion;
- If the number of “mountains” selected is greater than three, the three highest “mountains” are selected and then instructions for the previous point are followed.

**Figure 21:**
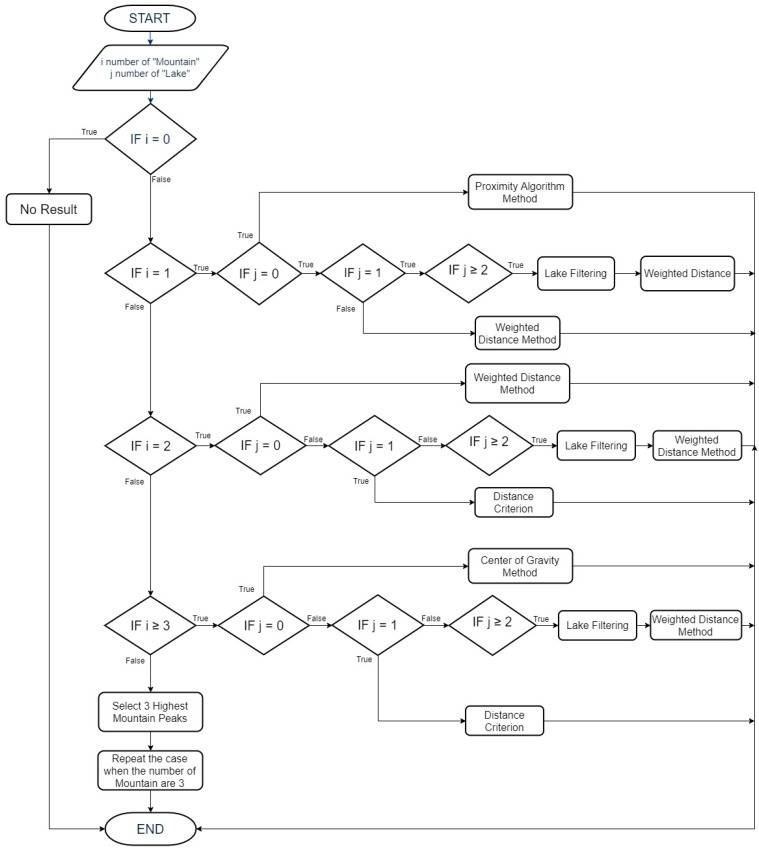
Flowchart of the positioning algorithm.

Figure 21 shows the positioning algorithm’s flowchart:

### 7.2. Distance Criterion

When there are two (or three) “mountains” and one “lake”, a pre-selection phase is activated through the distance criterion.

Suppose further that the “mountain” list is sorted in decreasing power, so that Montagna_0 is the highest power one.

- For a number of “mountains”, it is equal to three: if the selected “lake” is at a shorter distance from Montagna_0 than in Montagna_1 and Montagna_2, the method of weighted distance between the “lake” and the Montagna_0 is used;
- If only one “mountain” (e.g., Montagna_1) is at a shorter distance from Montagna_0, then P_Estimated_ is given by the midpoint between Montagna_0 and Montagna_1. If both “mountains” are at a shorter distance from Montagna_0, then P_Estimated_ is given by the center of gravity method”.

**Figure 22:**
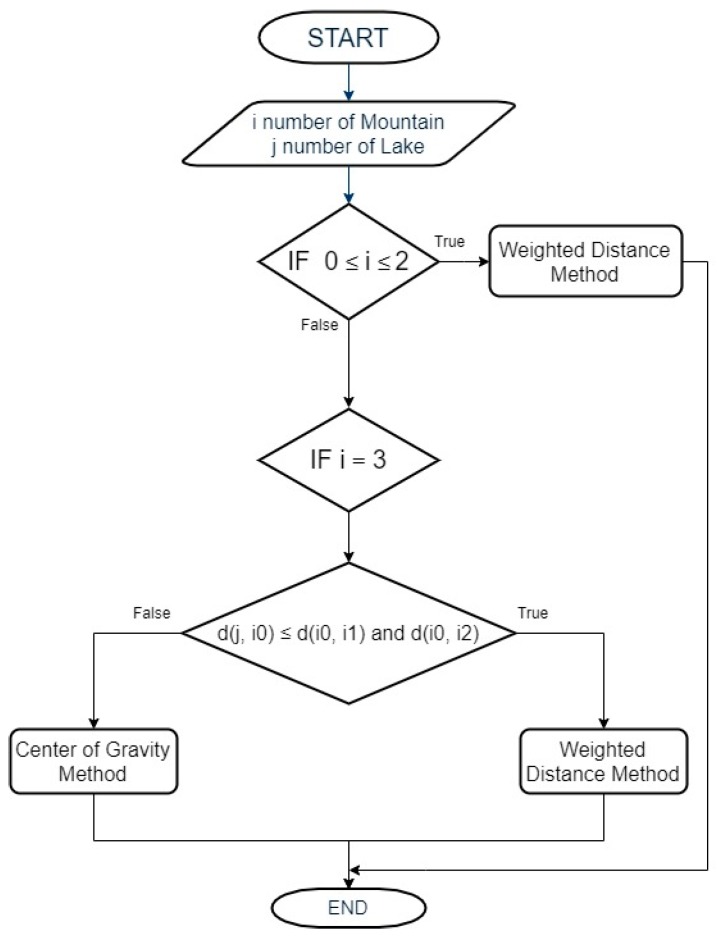
Flowchart of the distance criterion.

Figure 22 show the distance method’s flowchart:

### 7.3. Proximity Method

In general, an estimate of the position based on the proximity method uses a proximity sensor to detect the presence of nearby objects without any physical contact. In particular, the proximity sensor in this paper is represented by the femtocell, which measures the power levels received from a mobile terminal.

The proximity method consists in estimating the terminal at the position *P_M_* where there is the highest peak of power called the “peak of the mountain”.

(14) $$\{\begin{array}{c} P_{x}^{E}=\;P_{x}^{M}\\ P_{y}^{E}=\;P_{y}^{M}\\ P_{z}^{E}=\;P_{z}^{M} \end{array}\;,$$

(15) $$P_{Estimated}\left(P_{x}^{E},P_{y}^{E},P_{z}^{E}\right)=P_{M}\left(P_{x}^{M},P_{y}^{M},P_{z}^{M}\right)\;,$$

### 7.4. Weighted Distance Method

The weighted distance method is articulated in the following steps:

In order to process the data, first, we must look for all of the “mountains” and “lakes” on the surface.

Then, we can select the highest “mountain” peak.

- If no “mountain” is detected, no processing is possible and the routine is aborted;
- If no “lakes” are found, but there is at least one “mountain”, then the terminal is estimated in the position where the highest “mountain” peak lies;
- If only one “lake” is found, the position is estimated using the following formula:
  (16) $$P_{Estimated}=\delta_{M}*d_{LM}*\vec{u_{LM}},$$
  where $\vec{u_{LM}}$ is the directional versor that goes from the peak of the “lake” to the peak of the “mountain”.
- The more “lakes” are found, the less probable steps must be taken. To do this, the distance between the peak of the “mountain” (i.e., the highest power value) and the minimum point of each identified “lake” must be calculated and the one with the lowest $M_{n}$ value must be chosen, where $M_{n}$ is given by the following formula:
  (17) $$M_{n}=0.5\;*\left(\frac{D_{L_{i}-M}}{D_{Norm}}\right)+0.5\;*\left(\frac{L_{peak}}{P_{Norm}}\right),$$
  where $D_{L_{i}-M}$ is the distance between the *i*-th “lake” and the selected peak of the “mountain”, and is obtained by the following formula:
  (18) $$D_{L_{i}-M}=\|P_{L_{i}}-P_{M}\|,$$

Once a single “lake” is selected, proceed following the instructions outlined in point 2.

### 7.5. Center of Gravity Method

This method is applied when only three “mountains” are found. In particular, this consists of considering the three peaks of “mountain” as the three vertices of a triangle and calculating the center of gravity:

(19) $$\{\begin{array}{c} P_{x}^{E}=\;\frac{P_{x}^{M0}+P_{x}^{M1}+P_{x}^{M2}}{3}\\ P_{y}^{E}=\;\frac{P_{y}^{M0}+P_{y}^{M1}+P_{y}^{M2}}{3}\\ P_{z}^{E}=\;\frac{P_{z}^{M0}+P_{z}^{M1}+P_{z}^{M2}}{3} \end{array}$$

## 8. The TestBed Scenario

**Figure 23:**
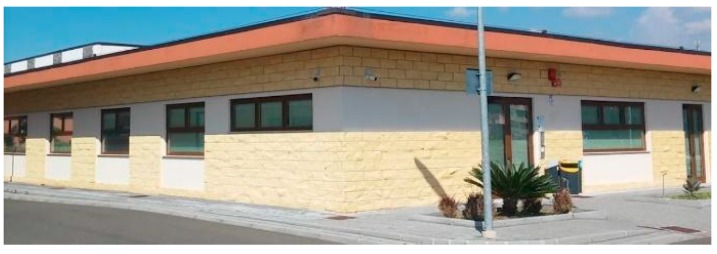
Technological pole building.

**Figure 24:**
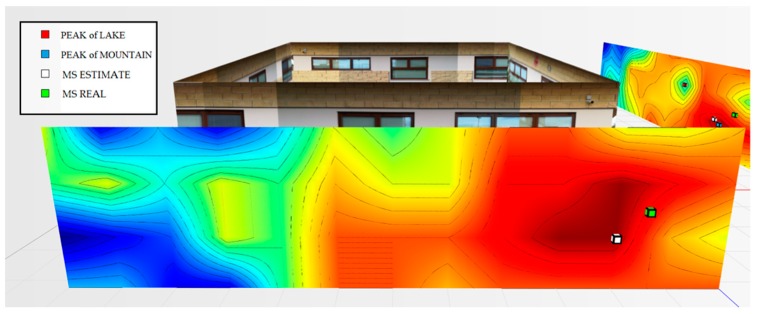
Power levels for wall 1 relative to the terminal located in the room on the left at 1.5 m.

**Figure 25:**
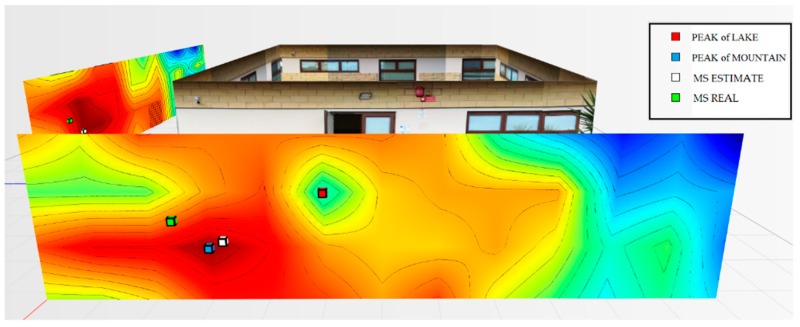
Power levels for wall 2 relative to the terminal located in the room on the left at 1.5 m.

For the testbed scenario, part of the technological center of the University of Catania was used during the algorithm validation phase. The choice was dictated by the possibility of having a structure characterized by different types of material such as concrete blocks, glass windows, external lava stone, wooden, and iron doors. Each material, as is known, has a different attenuation coefficient, and therefore it has been possible to simulate situations of isotropic and non-isotropic propagation, obtaining cases with mountains only (power peaks), but also in cases with the presence of lakes (signal sources’ RF that radiate laterally due to the high attenuation of the material along the direction orthogonal to the position of the mobile terminal to be geolocalized). In particular, for the geolocation tests, the mobile terminals were positioned inside the laboratory rooms along an external edge of the structure. The measures were carried out through a trestle adjustable in height where the femtocell was positioned, and considered a grid of 4 × 12 points equidistant to 1 m to cover the external walls. The data obtained from the power measurements were interpolated by obtaining images representing the so-called power walls used as input to the classification and geolocation algorithms described in the previous sections. In order to evaluate the effectiveness of the algorithm, two adjacent walls of the technological pole building of the University of Catania were considered (see Figure 23), and the terminal estimation algorithm was applied to the obtained data (Figure 24 and Figure 25).

It was decided to indicate the real position of the mobile terminal as a green cube, its estimated position in white, the peak of the “lake” in red, and, finally, the peak of a “mountain” in blue. This alternating choice of colors serves to discriminate, in the image, the position of the peak of the “mountain” and the peak of the “lake”. It is important to note that the alternating choice of colors does not affect the color that indicates the highest and lowest power. In fact, in the image, the area in red indicates a higher received signal strength than the blue zone.

Figure 24 shows the estimate of a “mountain” for wall 1. The terminal was at a height of 1.5 m and is represented by a green cube. It is important to point out that for all the tests conducted, the terminal was always placed in the same position and the measurement was read using an app (TeamViewer), with screen sharing on a PC desktop. This solution was introduced because the human body near the mobile terminal could make oscillations and variations in the received power.

The application of the terminal estimation algorithm, in this case, exploits the proximity method to estimate the terminal in the peak of power characterized by the “mountain”. The $P_{Estimated}$ was estimated to be 0.74 m away from the actual position.

As for wall 2 ( Figure 25), only one “lake” was found and a maximum peak of the “mountains”. In this case, the algorithm applied the weighted distance method between the “lake” and the “mountain”. The $P_{Estimated}$ was estimated to be 0.94 m away from the actual position.

Excellent localization accuracy could be observed in both cases.

Taking into consideration the second wall, if an operator is present, who evaluates the power wall derived from the values, the point where the terminal is likely to be is the peak power point identified as the “mountain”. To improve the localization estimate, therefore, it is possible to activate filters that allow, based on the assigned percentage, to take into account, or not, additional “mountains” or “lakes”. In particular, the percentage is set at 50% by default (i.e., it must exceed 50% of height/depth with respect to the maximum/minimum peak).

**Figure 26:**
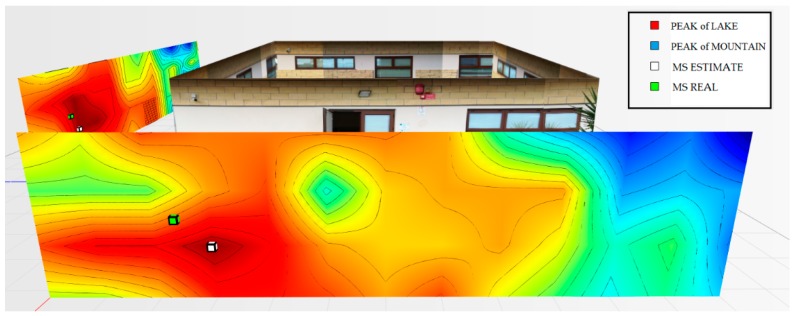
Power levels for wall 2 relative to the terminal located in the room on the left at 1.5 m with the filter.

If in this case, we apply a filter for the “lakes” of 70%, only a peak maximum power will be found. Therefore, the “proximity method” will be applied and the terminal will be estimated with an error of 0.78 m, with respect to the real position of the terminal. This can be observed in Figure 26 below.

**Figure 27:**
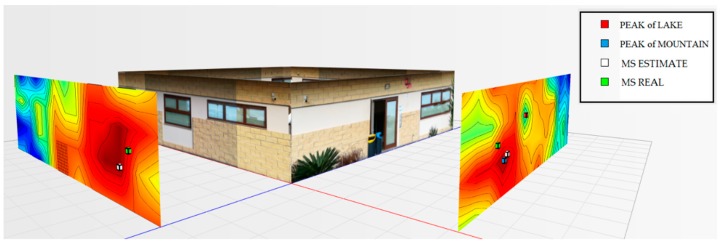
Overview of the power levels for walls 1 and 2, room terminal on the left at 1.5 m.

Figure 27 shows the overview of the two power walls relating to the terminal located in the room to the left at 1.5 m.

The second test was carried out by putting the terminal inside the room to the right (wall perspective 2) of the technological pole building, at a height of 1.4 m.

**Figure 28:**
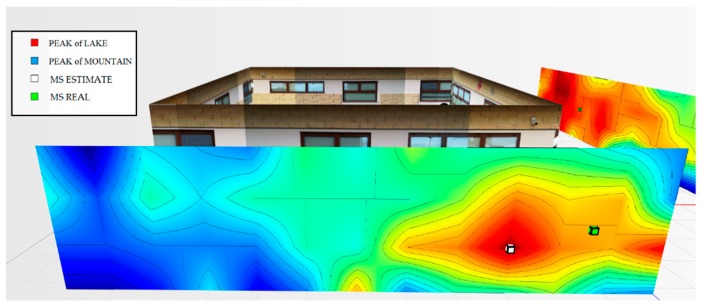
Power level for wall 1 related to the terminal located in the room to the right at 1.4 m.

Figure 28 graphically represents the power levels mapped on the two walls. Additionally, in this case, the new algorithm was applied. These results show that for wall 1, the algorithm used the proximity method and estimated the position of the device at the peak of the “mountain” at 1.6 m from the real position. Thus, a “lake” was found.

**Figure 29:**
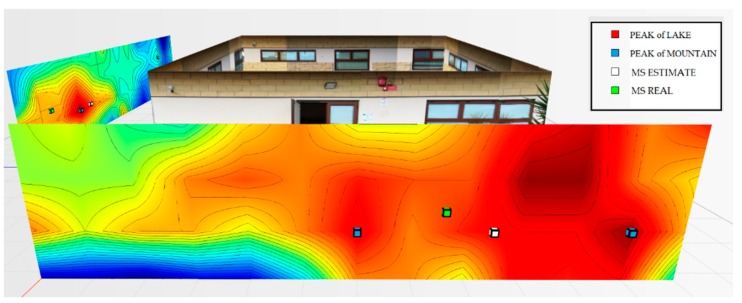
Power level for wall 2 related to the terminal located in the room to the right at 1.4 m.

As for wall 2 (Figure 29), two “mountains” and no “lakes” were detected. The algorithm used the distance criterion, estimating the terminal at 0.98 m away from the actual position.

**Figure 30:**
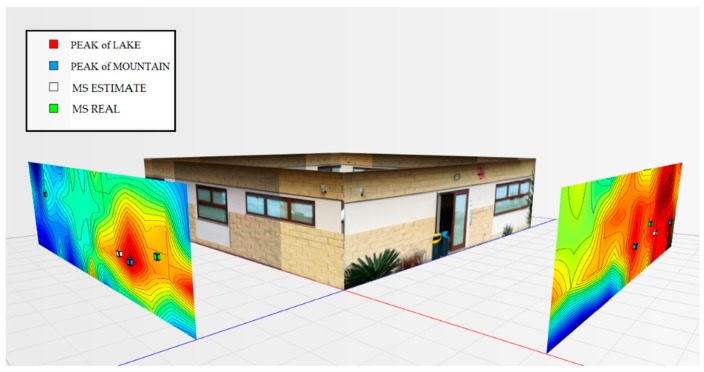
Overview of power levels for walls 1 and 2, room terminal to the right at 1.4 m.

In general, even the fourth test calls for excellent precision concerning the estimate of the terminal position. Figure 30 shows an overview of the two power walls relating to the terminal located in the room to the right at 1.4 m.

The proposed geolocation algorithm identified a mobile terminal through a 2D analysis of a power wall, but of course, it is possible to combine and aggregate the data to obtain a 3D geolocation that under certain circumstances can be decisive for the geolocation of a missing person.

## 9. Conclusions

In this paper, new classification and localization techniques for 4G terminals found under the rubble were defined; this study serves as the basis for a broader objective, namely the creation of an automatic platform to support civil protection in post-earthquake scenarios and, in general, scenarios where the collapse of a structure inhabited by people can occur. The proposed algorithm, thanks to the use of a femtocell on board a drone and 3D analysis algorithms of the measured radio signal power levels, allows for the rapid identification of the terminals possibly present under the rubble and, therefore, of any people situated nearby. Through the development of a simulation tool and a long series of field measurements, the performance of the algorithms proposed for the classification and localization phase was verified.

For the classification phase, by applying a “Safe Zone” of 10% (with respect to the short side of the monitoring area), it can be seen that for each monitoring area (of different sizes), there was an accuracy of 100%, regardless of the size of the area and the level of the rubble and, therefore, from the height of the drone. For the OUT terminals, the accuracy decreased as the level of the rubble increased and varied according to the size of the monitoring area from 80% to 60%.

With regard to the localization phase, the first results show that the exclusive use of the proximity method leads to an incorrect estimate of the terminal when it is under the rubble, or, in any case, behind obstacles of uneven distribution or made of different materials. In this regard, three different methods were devised which, combined under a single algorithm, allow for the effective identification of mobile terminals with errors on average of around 1 m, or errors that show that these techniques represent a useful and real support for civil protection actions.

## Figures and Tables

**Table 1:** LTE Femto AP–General H/W specification.

Title		Spec.	Note
Frequency Band	LTE	1805 ÷ 1880 MHz downlink	LTE Band 3
		1710 ÷ 1785 MHz uplink	
Centre Frequency		1849.9 MHZ DL 1754.9 MHz UL	1649 EARFCN DL 19649 EARFCN UL
Transmit Power		17 dBm/path	2 RF output types
Bandwidth		20 MHz FDD	
Antennas		2 × 2 MIMO	External Antenna (Gain: 3dBi)
Main Chipset	Baseband	Qualcomm FSM9905	SoC
	RF	Qulacomm FTR8900	RF
External Interface	Ethernet	Qualcomm AR8033 1 Gbps × 1 (Backhaul)	1000Base-T/RJ-45
Memory	RAM	2 GByte	DDR3L RAM
	ROM	4 GByte	Emmc
